# *Bletilla striata* Polysaccharide-Containing Carboxymethyl Cellulose Bilayer Structure Membrane for Prevention of Postoperative Adhesion and Achilles Tendon Repair

**DOI:** 10.1021/acs.biomac.4c00463

**Published:** 2024-06-27

**Authors:** Zhi-Yu Chen, Shih-Heng Chen, Shih-Hsien Chen, Pang-Yun Chou, Che-Yung Kuan, I-Hsuan Yang, Chia-Tien Chang, Yi-Chun Su, Feng-Huei Lin

**Affiliations:** † Department of Biomedical Engineering, College of Medicine and College of Engineering, National Taiwan University, Taipei 10617, Taiwan, ROC; ‡ Institute of Biomedical Engineering and Nanomedicine, National Health Research Institutes, Miaoli 35053, Taiwan, ROC; § Department of Plastic and Reconstructive Surgery, Chang Gung Memorial Hospital, Chang Gung University and Medical College, Taoyuan 33305, Taiwan, ROC; ∥ Institute of Molecular and Cellular Biology, College of Life Science, National Tsing Hua University, Hsinchu 300044, Taiwan, ROC

## Abstract

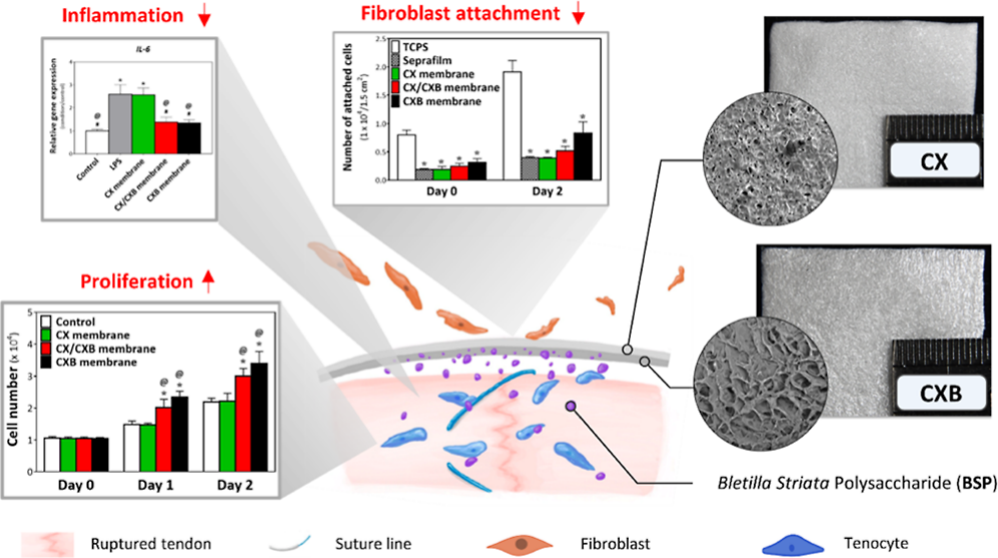

Postoperative tissue adhesion and poor tendon healing are major clinical problems associated with tendon surgery. To avoid postoperative adhesion and promote tendon healing, we developed and synthesized a membrane to wrap the surgical site after tendon suturing. The bilayer-structured porous membrane comprised an outer layer [1,4-butanediol diglycidyl ether cross-linked with carboxymethyl cellulose (CX)] and an inner layer [1,4-butanediol diglycidyl ether cross-linked with *Bletilla striata* polysaccharides and carboxymethyl cellulose (CXB)]. The morphology, chemical functional groups, and membrane structure were determined. In vitro experiments revealed that the CX/CXB membrane demonstrated good biosafety and biodegradability, promoted tenocyte proliferation and migration, and exhibited low cell attachment and anti-inflammatory effects. Furthermore, in in vivo animal study, the CX/CXB membrane effectively reduced postoperative tendon–peripheral tissue adhesion and improved tendon repair, downregulating inflammatory cytokines in the tendon tissue at the surgical site, which ultimately increased tendon strength by 54% after 4 weeks.

## Introduction

Tendons are connective tissues characterized by a brilliant white color and fibroelastic texture. They are composed of highly ordered dense fiber bundles [mainly type-I collagen (COL-I)]; thus, they have a high tensile strength to transmit the forces generated by muscle contraction to the bones.^1−3^ More than 30 million ligament and tendon injuries occur annually worldwide.^4^ Work-related incidents, accidents, sports, overuse, and age are important risk factors for tendon injuries. According to a recent report, 11–37 of every 100,000 middle-aged individuals suffer from acute Achilles tendon rupture every year. European yearly healthcare expenditure for tendon injuries exceeds €115 billion and that of the US is estimated at USD 30 billion.^5−8^

Immediate surgical reconstruction of the ruptured or lacerated tendon is the most common therapeutic modality.^9^ Postoperative adhesion (PA) and inadequate repair are two major possible clinical complications that can occur after the surgical repair of a tendon injury. One factor responsible for PA is the presence of exogenous fibroblasts at higher concentrations than endogenous tenocytes, caused by a prolonged inflammatory phase of the healing process. This overwhelming inflammatory response at the surgical site between the tendon and synovial sheath causes excessive fiber hyperplasia at the repair site.^10,11^ Patients with severe PA require additional surgeries for adhesiolysis, which not only increases the risk of infection but also increases the clinical burden and medical and health costs to the hospital.

Blood vessels play an important role in tissue healing by transporting inflammatory factors, cytokines, nutrients, and metabolites during the early and late healing stages. Tendons are hypovascular and hypocellular tissues that contain only a few tenocytes that are aligned in rows between the extracellular tendon matrix fibers. Postinjury traumatic tendon repair is typically poor and inefficient because of the inherent low cellularity and metabolic activity, as well as the insufficient mechanical strength acquired during the initial tendon healing that leads to rerupture.^12,13^ Previous studies have also shown that inflammation affects both the adhesion of peripheral tissues after surgery and tissue healing. In particular, dysregulated inflammation and altered macrophage phenotypes hinder tissue healing.^14,15^ Therefore, regulating the expression of inflammatory factors at the injured tendon surgical site to avoid excessive inflammation is important for reducing the occurrence of PA in peripheral tissues and promoting tendon repair.

Seprafilm and ADEPT are two commonly used antiadhesion barrier products approved and regulated by the US Food and Drug Administration (FDA).^16^ Carboxymethyl cellulose (CMC), a component of Seprafilm, is widely used in the pharmaceutical and food industries as an FDA-approved viscosity modifier, emulsifier, lubricant, and stabilizer for pharmaceutical dosage formulations. This material has excellent water-absorption, swelling, noncytotoxic, biocompatibility, and biodegradability properties.^17^ CMC is a component of many available antiadhesion and wound-dressing products, including IntraSite Gel and Purilon Gel. Although these products exhibit good antiadhesion properties in animal and clinical trials, they have a limited ability to reduce inflammatory factors and improve tissue repair.

The authors have previously reported that polysaccharides extracted from the traditional Chinese medicine *Bletilla striata* (BSP) can be used to replace growth factors such as bone morphogenetic protein 2 and platelet-rich plasma and stem cell-derived conditioned medium can effectively improve the proliferation and migration of human tenocytes and their ability to secrete collagen.^18^ BSP has anti-inflammatory and antioxidative properties. For instance, Chen et al.^19^ determined that BSP improved the climbing ability and survival rate of adult flies by decreasing the production of reactive oxygen species, increasing antioxidant enzyme activity, and inhibiting cell death. In 2023, Lin et al.^20^ confirmed that BSP combined with resveratrol effectively reduced inflammatory markers in early osteoarthritis (OA) in vitro and in preliminary lipopolysaccharide (LPS)-induced OA rat experiments. BSP is a water-soluble polysaccharide that can be directly added to the culture medium at specific concentrations for in vitro cell experiments. However, further animal experiments require a good carrier for stable release to delay its rapid metabolism in the body. CMC was selected in this study as the BSP carrier because of its good antiadhesion properties.

**Scheme 1:**
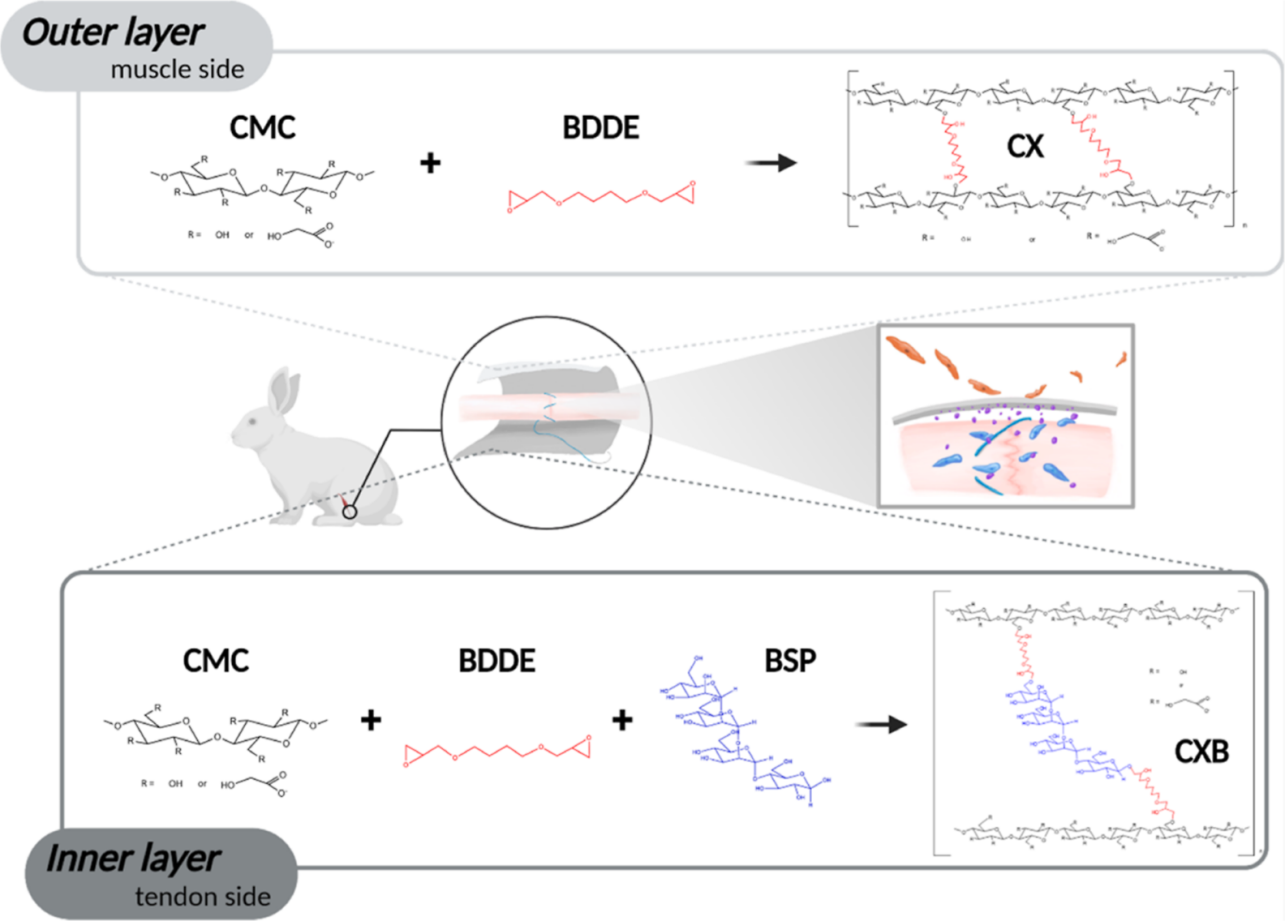
Schematic Illustration of the CX/CXB Bilayer Structure Membrane and Its Application as a Barrier to Prevent PA and Improve Tendon Repair The CX Layer Was Composed of 1,4-Butanediol Diglycidyl Ether (BDDE) Cross-Linked with CMC and the CXB Layer Was Composed of BDDE Cross-Linked with BSP and CMC

This study aimed to combine CMC, an antiadhesion material, with BSP, which has good anti-inflammatory, cell proliferation, and migration capabilities, to develop a porous membrane with a bilayer structure that can be wrapped around a sutured tendon. The authors hypothesized that the outer layer of the membrane would reduce fibroblast attachment to the membrane, thereby reducing tissue adhesion. The presence of an inner-layer membrane enables the sustained release of BSP to decrease inflammatory factors and enhance tenocyte proliferation and migration at the injured tendon site. Scheme 1 shows a schematic of the proposed bilayer structure membrane.

## Experimental Section

### Synthesis and Characterization of the Bilayer Structure Porous Membrane

Solution A: to prepare solution A, 2 g of CMC (medium viscosity, 400–800 cP, C4888, Sigma, USA) was added to 100 mL of ddH_2_O, followed by the addition of 0.01 mL of BDDE (liquid, 220892, Sigma, USA), and stirred for 8 h.

Solution B: to prepare solution B, 2 g of CMC and 0.25 g of BSP (previous publication of extraction method^18^) were added to 100 mL of ddH_2_O, followed by the addition of 0.01 mL BDDE, and stirred for 8 h. Solutions A and B were dialyzed (3.5 kDa cutoff, 60,035,515, Orange Scientific, Belgium) for 24 h. The viscosity and molecular weight (MW) of the intermediate gel after dialysis were measured using rheology (DHR-1, TA Instruments, Ltd.) and gel permeation chromatography (GPC; tested by Han Gene Technologies, Ltd.) analyses. 1 mL of solution A was poured into a 3.5 cm dish, air-dried for 24 h, and frozen in a freezer for 6 h at −20 °C. Thereafter, 1 mL of solution B was added to the same 3.5 cm dish, covering the layer of solution A, and frozen in a refrigerator for 6 h. The final CX/CXB bilayer porous membrane was obtained by lyophilization.

Fourier transform infrared (FT-IR; Spotlight 200i, PerkinElmer, USA) and nuclear magnetic resonance (NMR; 400-MR, Varian, USA) spectral analyses were performed to determine the presence of functional groups and chemical structure of the CX/CXB membrane. The membrane morphology was observed using a dissecting microscope (SMZ745T, Nikon, Japan) and scanning electron microscopy (SEM; TM-100, HITACHI, Japan).

### Swelling Ratio, Degradation, and BSP Release Profile of the CX/CXB Membrane

SR: 1 mg of the membrane was swollen in PBS at room temperature (RT, 25 °C). Excess PBS attached to the periphery of the membrane was removed at different time points (0–60 min) by placing it on filter paper, and the weight was measured and recorded.

Degradation: the membrane mass-to-PBS volume ratio was 10 mg:2 mL. The membranes were soaked in PBS at 37 °C. The specimens were obtained between 0 and 21 d, at which point, the liquid was removed and the sample was freeze-dried and weighed. The variation in weight of the membrane was calculated to obtain its degradation curve.

The BSP release profile of the CX/CXB membrane was analyzed using the anthrone test. 1 mL of PBS was added to 10 mg of the membrane in a microtube and then incubated at 37 °C under continuous shaking for 0–21 d. The samples were extracted and mixed with a 0.2% anthrone (52445, Sigma, USA) solution. Thereafter, the absorbance at 625 nm was measured using an enzyme-linked immunosorbent assay (ELISA) reader (SpectraMax Plus384, Molecular Devices, USA).

### Proliferation Test

Rabbit Achilles tenocytes (RATs) were seeded in 24-well cell culture plates in Dulbecco’s modified minimal essential medium (DMEM; high glucose, D5671, Sigma, USA) using a previously published isolation method.^3^ The membranes were placed on top of a Transwell plate and cocultured with RATs. Cell numbers were calculated using a hand-held automated cell counter (Scepter 2.0 Cell Counter, MERCK Millipore).

### Migration Analysis

RATs were seeded in the upper Transwell chambers with 200 μL of DMEM, and the lower chambers contained 500 μL of DMEM with a different type of membrane.^21,22^ Those that migrated to the lower side of the membrane were stained with ActinGreen (R37110, Thermo, USA) and then fixed on slides using DAPI-mounting medium (H-1200, VECTOR, USA). The prepared slides were observed and photographed using a fluorescence microscope.

### Gene Expression of Inflammatory Cytokines in LPS-Induced RATs

RAT-induced inflammation occurred after treatment with 100 ng/mL LPS for 6 h. The medium was removed and cocultured with membranes for 6 h. RATs were then collected, and the total amount of RNA was extracted using TRIzol reagent. Real-time polymerase chain reaction (RT-PCR) was performed using a KAPA SYBR FAST One-Step (KK4650, Roche, Switzerland) kit with the following inflammation-related gene primers: *GAPDH*, *IL-1β*, *TNF-α*, and *IL-6*. *GAPDH* was used as an internal control. The relative gene expression was quantified using the 2^–ΔΔ*Ct*^ method. Table 1 lists the primer sequences used.

**Table 1:** Rabbit-Specific Primers Were Used for the RT-PCR of Inflammatory Genes

gene		primer sequence (5′ to 3′)
*IL-1β*	forward	TTGAAGAAGAACCCGTCCTCTG
	reverse	CTCATACGTGCCAGACAACACC
*IL-6*	forward	CTACCGCTTTCCCCACTTCAG
	reverse	TCCTCAGCTCCTTGATGGTCTC
*TNF-α*	forward	CTGCACTTCAGGGTGATCG
	reverse	CTACGTGGGCTAGAGGCTTG
*GAPDH*	forward	GGGTGGTGGACCTCATGGT
	reverse	CGGTGGTTTGAGGGCTCTTA

### Cell Attachment Analysis of Membranes

Human dermal fibroblasts (HDFs), isolated from human scar tissue (Cheng Gung Memorial Hospital, IRB ref: 201601422B0), were seeded onto the surface of a prewet membrane (diameter of 1.4 cm) in 24-well plates and then incubated at 37 °C for 4 h to allow adhesion of cells to the membrane. The cells were then transferred to a new 24-well plate. The number of attached HDFs was quantified using a WST-1 assay (MK400, Takara, Japan). The absorbance was measured at 450 nm using an ELISA reader.

### Animal Study Using the Tendon Rupture and Repair Model

Animal experiments and procedures were performed in accordance with the Guide for the Care and Use of Laboratory Animals issued by the Animal Research Committee of the Chang-Gung Memorial Hospital (no. 2021071902), which was followed by the National Research Council’s Guide for the Care and Use of Laboratory Animals. Male New Zealand white rabbits were randomly assigned to the normal, suture (only surgical suture without wrapping any membranes), Seprafilm, and CX/CXB bilayer porous membrane groups. All results were obtained from six independent experiments. The Achilles tendon of the rabbit leg was cut. Thereafter, the ruptured tendon was sutured and wrapped with Seprafilm or the CX/CXB bilayer porous membrane (the CXB layer was situated on the inner tendon side and the CX layer was on the outer muscle side) at the suture location. Finally, the wound on the lower leg of the rabbit was closed, antibiotics were administered to prevent wound infection, and the ankle was immobilized with a cast. The rabbits were euthanized 4 weeks postoperatively by inhaling excess CO_2_, and the Achilles tendons were harvested.

The Achilles tendon tissue was fixed in formalin, and histological sections were stained with hematoxylin and eosin (H&E) and Gomori’s trichrome (24205, Polysciences, USA). The breaking force of the repaired tendons was measured using an electromechanical test system (model 42, MTS criterion). The Achilles tendon tissue was extracted using a tissue bead crusher homogenizer (μT-12, TAITEC, Japan) after adding tissue protein extraction reagent (T-PER, 78510, Thermo, USA).

The inflammatory factors affecting the Achilles tendon tissue were analyzed using commercially available kits, namely, *IL-1β* (MBS2024461, MyBioSource, USA), *IL-6* (DY7984, R&D Systems, USA), and *TNF-α* (DY5670, R&D Systems, USA). The extracellular matrix (ECM) of the Achilles tendon tissue was analyzed using a hydroxyproline assay kit (K226-100, Biovision, USA) and Western blotting (WB), including COL-I (MA1-26771, Thermo, USA), type-III collagen (COL-III) (MA1-22147, Thermo, USA), Decorin (DCN; ab181456, abcam, UK), Biglycan (BGN) (ab226991, abcam, UK), Tenomodulin (TNMD) (ABIN2783853, Antibodies, Germany), and beta-actin (ab8227, abcam, UK).

### Statistical Analysis

All data are expressed as the mean ± standard deviation of at least three independent experiments. Statistical differences between groups were tested using Student’s *t*-test, one-way analysis of variance post hoc tests, and Tukey’s test using GraphPad Prism software (GraphPad Software, USA). A probability (*p*) value less than 0.05 (*p* < 0.05) was considered statistically significant.

## Results

### FT-IR and NMR Analyses of the Membranes

BDDE, which is FDA-approved and biofriendly, was selected in this study as the cross-linker for CMC and BSP to slow material degradation. Rheology and GPC were initially used to analyze the viscosity and MW of the developed gels after the BDDE was cross-linked and dialyzed. The results of this analysis, listed in Table 2, revealed that the BDDE-cross-linked CMC (CX) and CMC–BSP (CXB) (*p < 0.05) exhibited an increase in both the viscosities and MWs.

**Table 2:** Viscosity and MW of the Developed Gels^a^

	viscosity (mPa·s)	MW (kDa)
CMC	251.20 ± 1.68	174.72
BSP	1.43 ± 0.35	65.06
CX	292.23 ± 8.01*	230.25
CXB	365.78 ± 1.10*	214.62

a *p < 0.05; *n* = 3.

**Figure 1:**
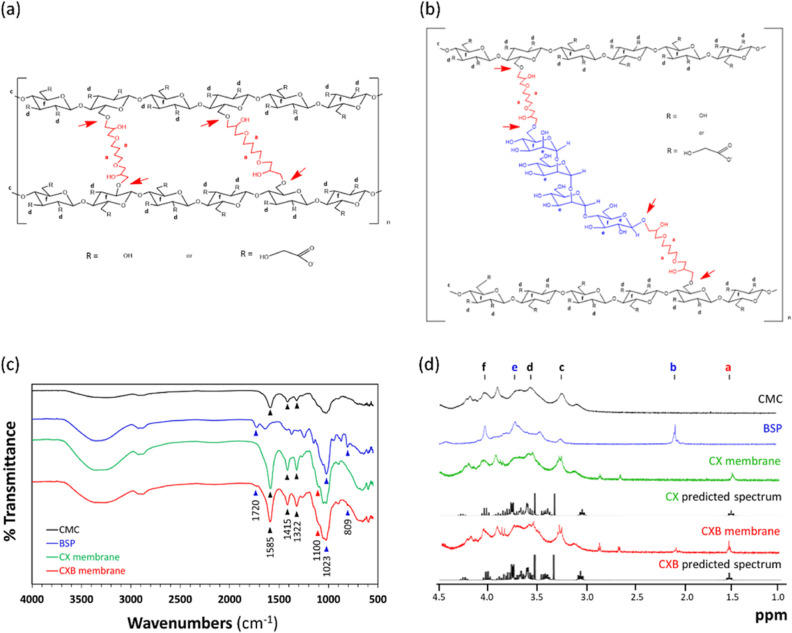
Estimated structures of the (a) BDDE cross-linked CMC (CX) and (b) BDDE cross-linked BSP and CMC (CXB). CMC, BSP, and BDDE are represented in black, blue, and red, respectively. The functional group composition and structure of the membrane were determined using (c) FT-IR and (d) ^1^H NMR.

FT-IR and NMR were used to more comprehensively analyze CX and CXB to determine the presence of functional groups and the overall structure of the membranes prepared from the above preliminary analysis gels. Figure 1a,b shows the simulated structures of the proposed CX and CXB, respectively. Black, red, and blue represent the CMC structure, the cross-linking agent BDDE, and the BSP structure, respectively. Figure 1c shows the FT-IR fingerprint spectra of the membranes (500–4000 cm^–1^). The absorption bands at 1322, 1415, and 1585 cm^–1^ were attributed to CMC, whereas those at 1023 and 1720 cm^–1^ were attributed to BSP. Characteristic pyranose peaks (809 cm^–1^) were also observed in the BSP and CXB groups. The epoxide groups at the two ends of the BDDE molecule preferentially reacted with the most accessible primary OH groups in CMC and BSP, forming a C–O–C ether bond (red arrow in Figure 1a,b). Therefore, the characteristic absorption peak of C–O–C was observed at 1100 cm^–1^ in the FT-IR spectra of the CX and CXB groups.

The ^1^H NMR spectra (Figure 1d) of the CX and CXB groups were similar to the predicted simulation spectra. The peak at 1.51 ppm (red “a” mark) was attributed to BDDE, the peaks at 2.08 and 3.73 ppm (blue “e” marks) were attributed to BSP, and the peaks at 3.30 and 3.58 ppm (black “c” and “d” marks, respectively) were attributed to CMC. Because the chemical structures of CMC and BSP both contain a hexose structure, the characteristic peak of hexose at 4.00 ppm (“f” mark) was also observed in the CMC, BSP, CX, and CXB groups.

### Characterization of the CX/CXB Bilayer Porous Membrane

**Figure 2:**
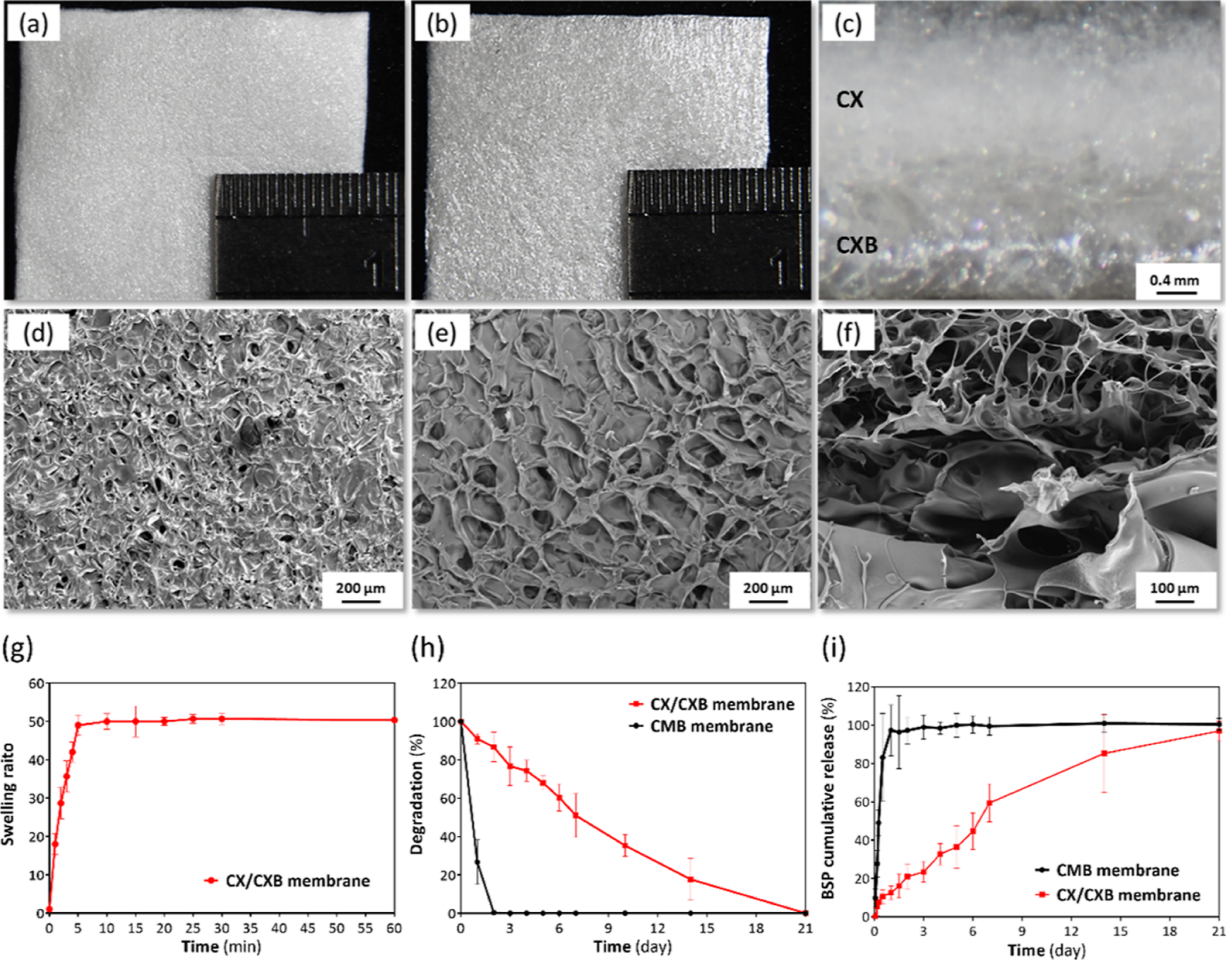
Characteristics analysis of the CX/CXB bilayer structure membrane. Morphological features of the (a) CX front, (b) CXB back, and (c) side of the membrane observed using a dissecting microscope. Membrane microstructure of the (d) CX front, (e) CXB back, and (f) cross-section of the membrane observed using SEM (g) swelling, (h) degradation, and (i) BSP cumulative release profiles of the CX/CXB membrane. CMB: CMC mixed with BSP.

After confirming that the materials of the muscle-side outer layer (CX) and tendon-side inner layer (CXB) membranes were successfully cross-linked, a CX/CXB bilayer-structured porous membrane was prepared using mold casting. The morphology of the CX/CXB membranes was observed using a dissecting microscope. The outer layer of the CX/CXB membrane was matte-white in color (Figure 2a). The inner side of the membrane was also white (Figure 2b) and brighter in color and smoother than the outer layer. Figure 2c shows a cross-section of the CX/CXB membrane. SEM was used to further observe the microstructure of the CX/CXB membrane. The CX/CXB membrane exhibited a porous structure at the CX in front (Figure 2d), the CXB at the back (Figure 2e), and even from the sides (Figure 2f). The membrane had pore sizes of 20–50 nm on the front CX side and 50–200 nm on the back CXB side.

Figure 2g shows the experimental swelling results for the CX/CXB membrane. The membrane absorbed a significant amount of water during the first 5 min of the experiment, reaching a maximum value 50 times than that of the original weight. Subsequently, the membrane ceased to absorb water. The BSP content in the solution was analyzed, and the weight of the residual membrane was measured at different time points to assess the degradation and release of the CX/CXB membrane in vitro. Figure 2h shows the in vitro degradation results. The CMB membrane (CMC mixed with BSP) completely degraded on day 2 of the experiment. In contrast, the CX/CXB membrane degraded slowly over 21 days. Figure 2i shows the cumulative amount of BSP released from the CX/CXB membrane. In summary, the uncross-linked CMB membrane released 100% of the BSP within 2 d of the experiment, whereas the CX/CXB bilayer structure porous membrane stably released BSP for 21 days.

### Cytotoxicity Analysis of the CX/CXB Membrane

**Figure 3:**
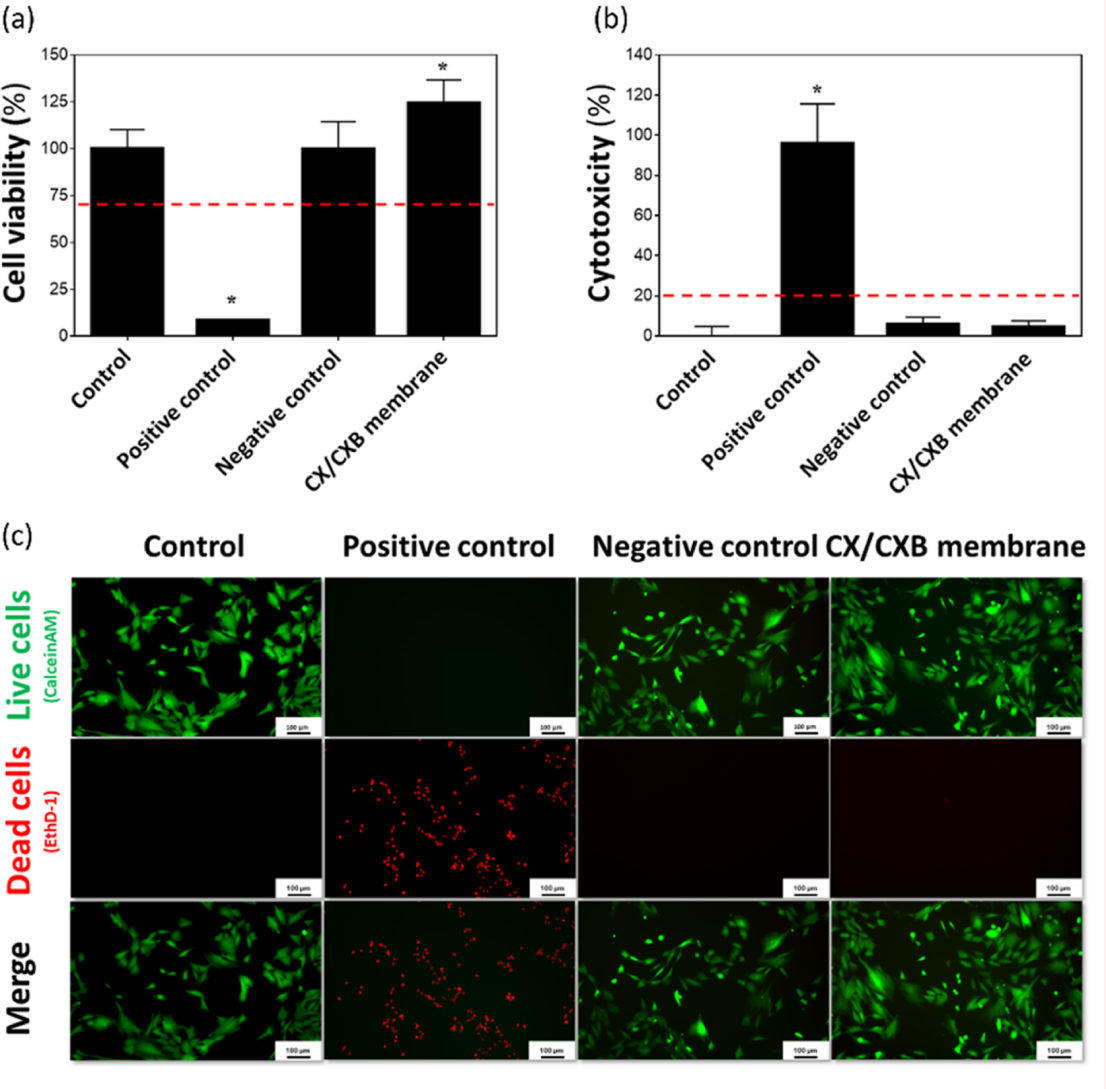
Cytotoxicity analysis of the CX/CXB membrane were examined by (a) WST-1, (b) LDH, and (c) live/Dead stain on RATs. *p < 0.05 compared with the control group. Positive control: ZDEC; negative control: Al_2_O_3_. *N* = 6.

The WST-1 results (Figure 3a) showed that the CX/CXB membrane developed in this study exhibited good cell viability (>70%) in RATs. A lactate dehydrogenase assay (Figure 3b) confirmed that the membrane had no obvious cytotoxicity (less than 20%). Additionally, qualitative live/dead fluorescent staining experiments (Figure 3c) revealed few or no red fluorescent signals in the CX/CXB membrane group, indicating that the membrane was noncytotoxic. The positive and negative control groups were treated with zinc diethyldithiocarbamate and Al_2_O_3_, respectively.

### Proliferation and Migration Analyses of the Developed Membranes

**Figure 4:**
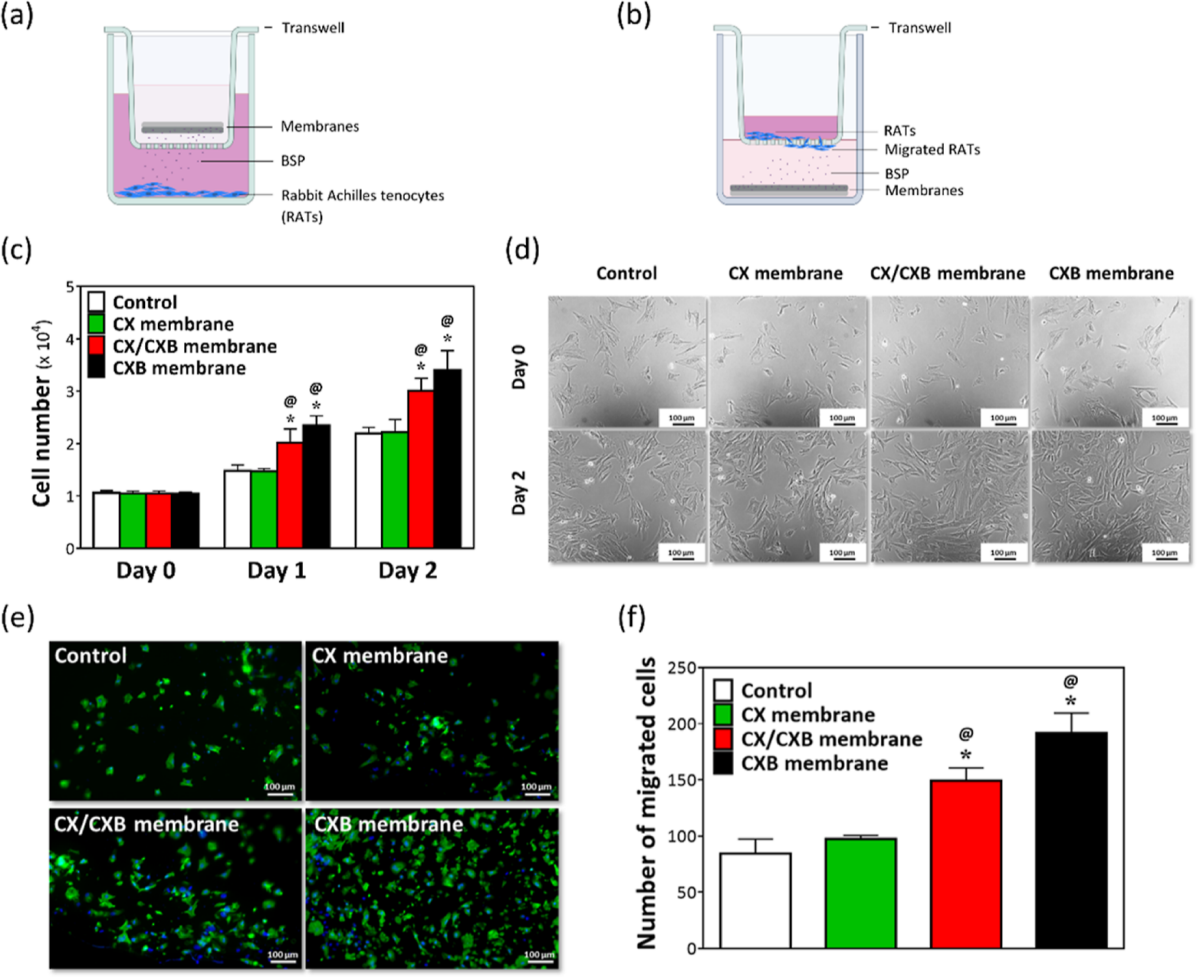
Functional tests of the CX, CX/CXB, and CXB membranes. (a) Schematic illustration of RATs cocultured with various membranes for 0, 1, and 2 d using Transwell. (c) Cell numbers and (d) photomicrograph results of the proliferation analysis. Migration analysis of the RATs cocultured with varying membranes for 24 h. (b) Schematic illustration of the Transwell migration test. (e) Fluorescent staining and the (f) corresponding quantitative migrated cell results, determined using ImageJ software. *p < 0.05 when compared with the control group and ^*@*^*p* < 0.05 when compared with the CX group. *N* = 6. Beta-actin and DAPI are shown in green and blue, respectively.

In the cell proliferation experiment, various membranes (CX, CX/CXB, and CXB) were individually placed in Transwell inserts. The inserts were then placed in the wells where the RATs were preseeded (Figure 4a). Figure 4c shows the counted cell numbers, and Figure 4d shows the bright-field images of the proliferated RATs. The growth trend and daily cell numbers of the CX group were similar to those of the control group. The number of RATs in CX/CXB and CXB, both of which contained BSP, increased significantly, as compared with that of the control group (*p < 0.05), after 1 d of coculture with the membranes.

Transwell plates were also used to coculture membranes and RATs to evaluate the ability of the CX/CXB membranes to attract cells toward the membranes (Figure 4b). Figure 4e presents the results of the fluorescent staining, and Figure 4f shows the quantitative results of the fluorescent images obtained after cell counting using ImageJ software. In the control and CX groups, only a few RATs migrated to the bottom of the Transwell insert. However, more RATs migrated in the CX/CXB and CXB groups than those in the control group (*p < 0.05).

### Anti-inflammatory Effects and Cell Attachment Tests of Various Membranes

**Figure 5:**
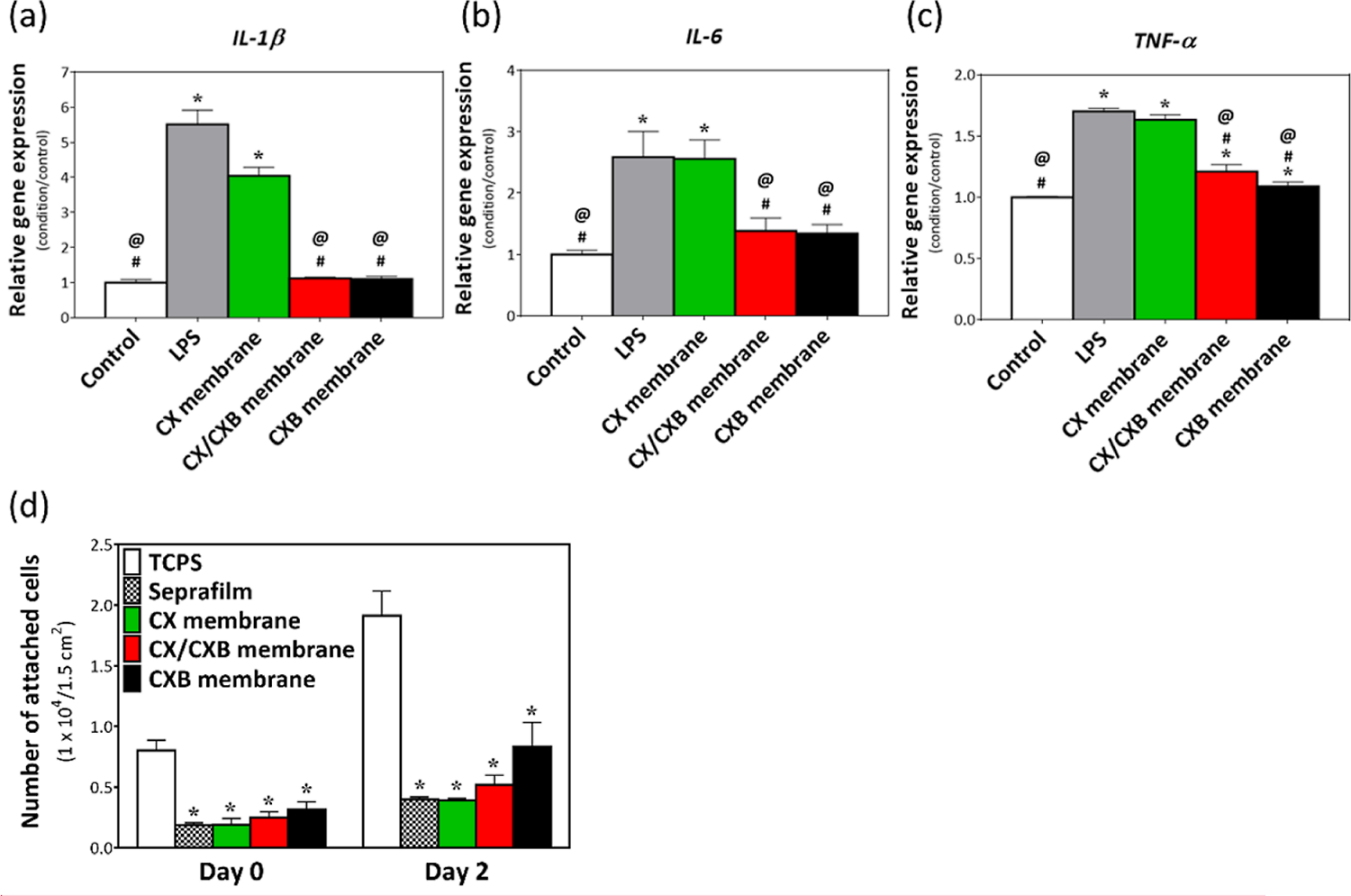
Gene expression of (a) *IL-1β*, (b) *IL-6*, and (c) *TNF-α* in RATs after LPS-induced inflammation (6 h) and coculturing with various membranes for 6 h. (d) HDF attachment test of various membranes, determined using WST-1 for 0 and 2 d. *p < 0.05 when compared with the control group. ^#^*p* < 0.05 when compared with the LPS group, and ^@^*p* < 0.05 when compared with the CX group. *N* = 3.

The gene expression results for the different membranes cocultured with RATs that induced LPS inflammation are shown in Figure 5a–c. The *IL-1β*, *IL-6*, and *TNF-α* gene expression levels in the LPS group were significantly higher than those in the control group (*p < 0.05), indicating that RATs successfully used LPS to induce inflammation. The inflammatory genes *IL-1β* and *IL-6* were significantly downregulated to baseline levels in the CX/CXB and CXB groups of the membranes cocultured with LPS-treated RATs (^#^*p* < 0.05). Although the expression of the *TNF-α* gene did not revert to the control level in CX/CXB or CXB cocultured with LPS-treated RATs, it was still significantly lower than that in the LPS group (^#^*p* < 0.05).

Cell attachment experiments using HDFs were performed to investigate whether the membranes developed in this study reduced cell adhesion. Figure 5d shows that the Seprafilm, CX, CX/CXB, and CXB membranes had only a few HDFs attached to them, as compared with the tissue culture polystyrene (TCPS) group on day 0. Although a few HDFs adhered to and grew on the membrane in the CXB group on day 2, the results were still significantly different (*p < 0.05) from those observed in the TCPS group. Therefore, the membranes developed in this study had similar anticell adhesion properties as Seprafilm.

### In Vivo Animal Study of CX/CXB Membranes

**Figure 6:**
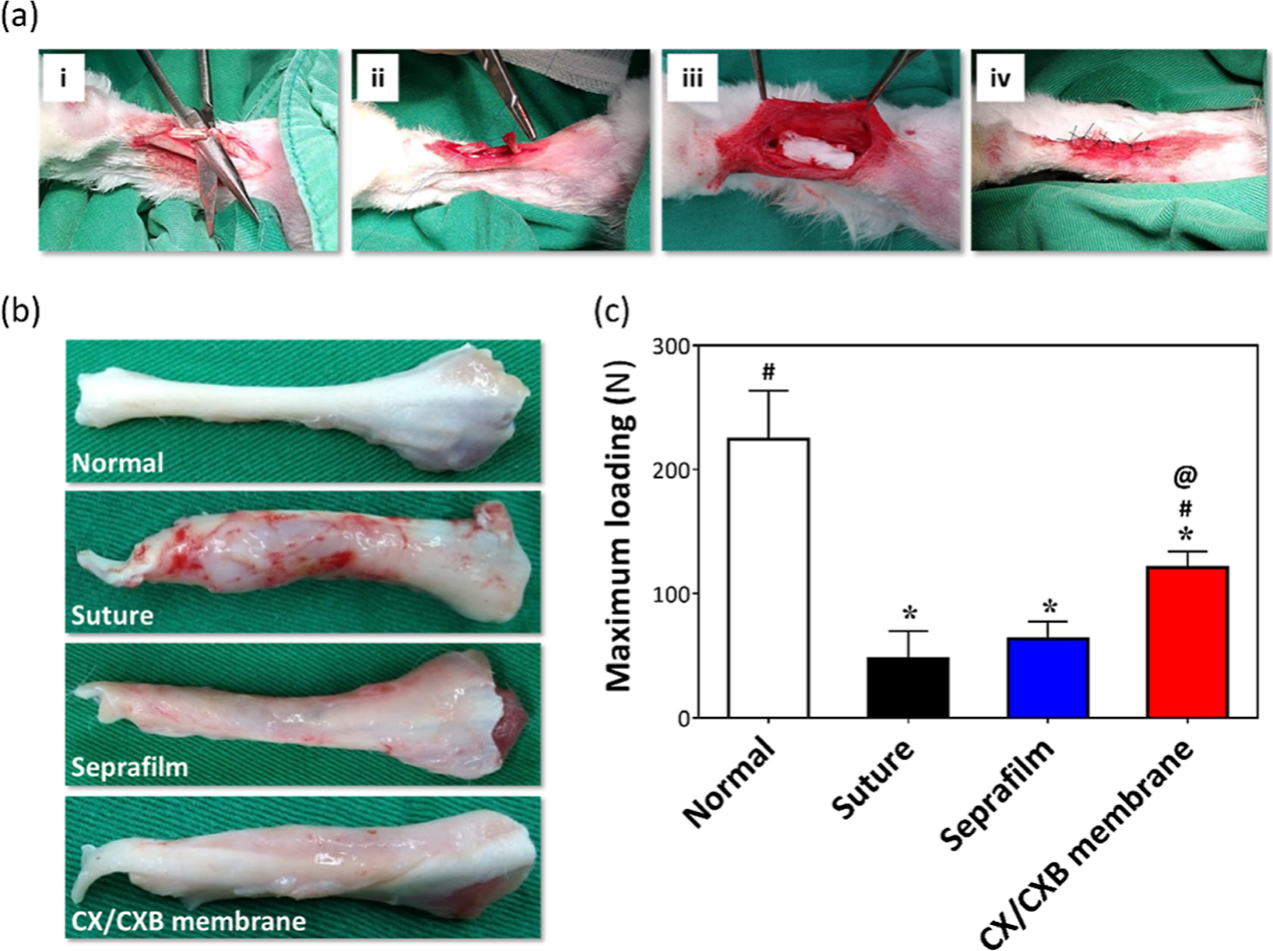
In vivo study of rabbit TRR. (a) Photographic images of the surgery process. The Achilles tendon was transected (i), the ruptured tendon was sutured using 5–0 prolene (ii), the CX/CXB bilayer porous membrane was wrapped around the surgical site (iii), and the skin was closed (iv). (b) Photographs and (c) tensile strengths of tendon tissues collected 4 weeks after surgery. *p < 0.05 when compared with the normal group, ^#^*p* < 0.05 when compared with the suture group, and ^@^*p* < 0.05 when compared with the Seprafilm group. *N* = 6.

New Zealand white rabbits were selected as the experimental animals for the in vivo study. The experiments were performed using a rabbit TRR model. Figure 6a shows the surgery process of the animal study. Figure 6b shows that the normal tendons were bright white with tightly packed tendon fibers. Dense adhesions caused by severe inflammation were observed in the suture group. Numerous adhesive and granulation tissues were observed around the tendon in the suture group but fewer in the Seprafilm and CX/CXB membrane groups, as compared with the normal group.

Figure 6c shows the breaking forces of the tendons; the maximum loadings of the normal tendon, suture, Seprafilm, and CX/CXB membrane groups were 225, 47, 63, and 121 N, respectively. The tendon breaking force in the Seprafilm group indicated that the film had a limited ability to repair the tendon despite preventing adherence to the surrounding tissue. Although the CX/CXB membrane group in this study also failed to restore the ruptured tendon strength to that of the normal group (54% of normal) through BSP stimulation, significant recovery was observed, as compared to the suture (^#^*p* < 0.05) and Seprafilm (^@^*p* < 0.05) groups.

### Evaluation of the Effect of Tendon Repair

**Figure 7:**
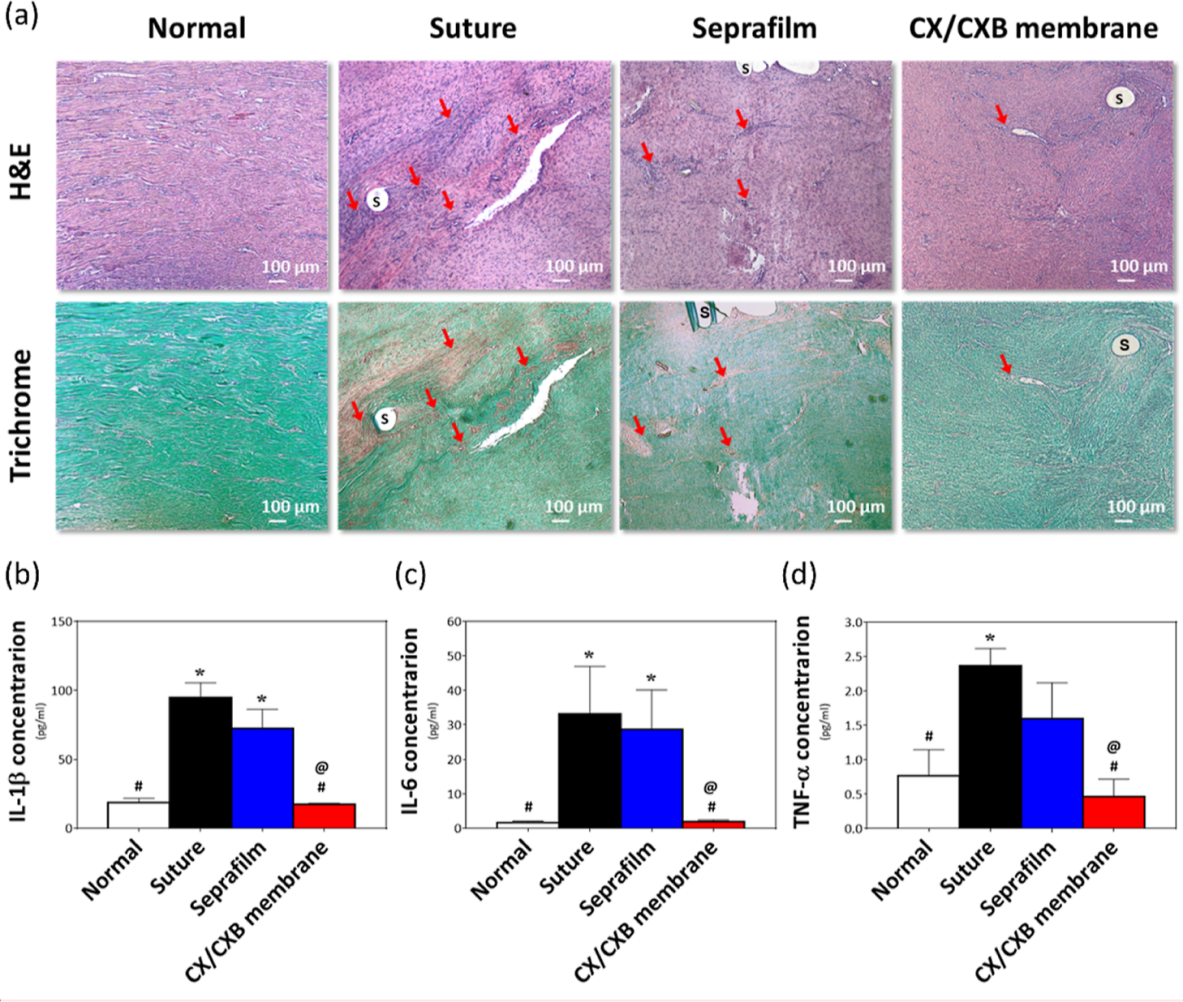
(a) H&E and Gomori’s trichrome stains of the repaired tendons. Inflammatory cells: red arrows, suture: S, nuclei: purple (HE) and black (trichrome), cytoplasm: red, and collagen: green. The (b) *IL-1β*, (c) *IL-6*, and (d) *TNF-α* inflammatory cytokines of the rabbit tendon were examined using ELISA. *p < 0.05 when compared with the normal group, ^#^*p* < 0.05 when compared with the suture group, and ^@^*p* < 0.05 when compared with the Seprafilm group. *N* = 6.

Tissue sections of the tendons were stained with H&E and Gomori’s trichrome stains (Figure 7a). The CX/CXB membrane group exhibited more organized and aligned collagen fibers with less inflammatory cell infiltration (red arrow) than the suture group; several inflammatory cells were also observed in the Seprafilm group. Incomplete tendon repair (not fully closed and still damaged) was observed in both the suture and Seprafilm groups.

### Quantification of Inflammatory Factors in Rabbit Tendons

The inflammatory cells observed in the repaired tendon tissues were further analyzed. The tendons were extracted to examine the variation in inflammatory cytokines using *IL-1β* (Figure 7b), *IL-6* (Figure 7c), and *TNF-α* (Figure 7d) ELISA kits. Only baseline levels of *IL-1β*, *IL-6*, and *TNF-α* were found in the normal tendon tissue. In contrast, the inflammatory cytokine levels increased significantly in the suture group (*p < 0.05). No statistical difference in the *IL-1β* and *IL-6* levels was observed in the Seprafilm group, as compared with that in the normal group; however, the expression of the overall inflammatory factors tended to decrease. The inflammatory factors in the CX/CXB membrane group were significantly downregulated to baseline expression levels.

### ECM Composition in the Repaired Tendon of a Rabbit

**Figure 8:**
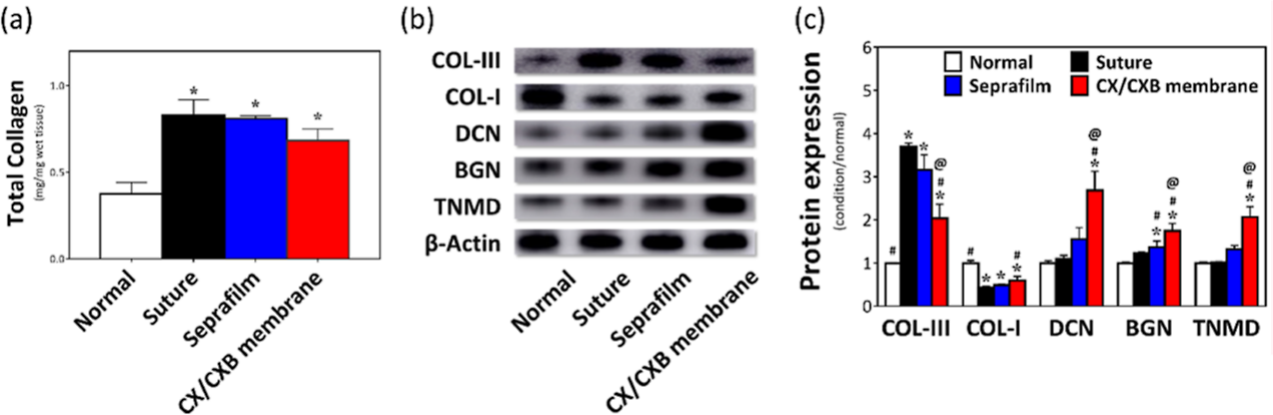
ECM analysis of the tendon tissues. (a) Total collagen content of the repaired tendons. (b) Western blot showing the COL-III, COL-I, DCN, BGN, and TNMD proteins. (c) Quantification of the WB bands. *p < 0.05 when compared with the normal group, ^#^*p* < 0.05 when compared with the suture group, and ^@^*p* < 0.05 when compared with the Seprafilm group. *N* = 6.

Figure 8a shows the total collagen content of the tendons. Although the total collagen contents of the suture, Seprafilm, and CX/CXB membrane experimental groups were higher (*p < 0.05) than that of the normal group, there was no statistically significant difference among the three groups. Therefore, WB was used to further analyze the various ECM proteins. Figure 8b,c shows the WB results. The collagen composition of the normal tendon tissue was COL-I and a small amount of COL-III, whereas the suture and Seprafilm groups contained large amounts of COL-III. A significant decrease in the expression of COL-III, as compared with that of the suture group (^#^*p* < 0.05), was observed in the CX/CXB membranes; an increase in the expression of COL-I was also observed. In vitro cell experiments confirmed that BSP increases tenocyte proliferation and migration. Therefore, numerous tenocyte-specific marker proteins (DCN, BGN, and TNMD) can be expressed in the CX/CXB membrane group.

## Discussion

Similar to other tissues, the healing process in tendons is divided into three overlapping phases: inflammatory, proliferation, and remodeling. However, the tendon remodeling stage often takes more than a year, whereas skin tissue heals within a few months. The complications of PA between the tendon and surrounding tissue will further delay repairs of the tendon.^23−25^ Barrier is one of the common ways to prevent PA. Early studies used hyaluronic acid (HA), alginate, and chitosan (CA), separately or in complexes, in the form of hydrogels, films, or fibers, as physical barriers to reduce the PA of the tendon.^26−29^ These studies demonstrated good antiadhesion effects in animal and clinical studies. However, complete restoration of the biomechanical properties of repaired tissue was difficult. The provision of a simple physical barrier, such as a sheath, to prevent PA may negatively influence tendon healing because tendon healing requires synovial fluid for nutrition.^30^ Therefore, ideal strategies require the design of multilayered materials with good porosity and anti-inflammatory factors to enhance the tissue regeneration factors of loaded materials to prevent PA without affecting tendon healing.

In 2014, Jiang et al.^31^ used a TGF-β3-loaded porous CA scaffold to mimic the synovial sheath of a tendon for antitissue adhesion. Jiang et al.^32^ developed a multilayer porous membrane using a celecoxib-loaded poly(l-lactic acid)–polyethylene glycol (PELA) fibrous membrane (outer layer), HA gel (middle layer), and PELA electrospun fibrous membrane (inner layer). In vivo animal experiments showed that this multilayer membrane had good antipostoperative tissue adhesion and did not affect the repair of the inner tendon when HA was used as the buffer, lubricant, or nutrient. Although many studies have shown that materials such as HA or synthetic polymers (polycaprolactone and polyurethane) have good antiadhesion properties,^33−36^ CMC was selected as the main antiadhesion material in this study because of its previously reported good antiadhesion properties and because it is easy to obtain, inexpensive, and easy to process and modify. Reports have even confirmed that the proliferation of cells on bilayered hydroxypropylmethylcellulose (HPMC)-CMC-coated substratum (CEL) was significantly decreased, and cells were arrested in the G1 phase and underwent apoptosis.^37^ In the cell attachment test of this study, it was also observed that materials containing CMC (even Seprafilm) have good anticell attachment properties.

An increasing number of studies have recently suggested that inflammatory cytokines (*IL-1β*, *TNF-α*, and *IL-6*) are involved in tendon repair and post-traumatic inflammatory responses that affect PA and healing. Exogenic cytokine sources are blood-derived leukocytes that migrate to the injured tendon owing to hemorrhage during the early inflammatory period. However, if the expression of inflammation-related cytokines is unbalanced at this stage, the inflammation period will be prolonged with the formation of hematoma, resulting in tissue adhesion.^38,39^ Significant granulation tissue was observed in the suture group due to hematoma (Figure 6b) in this study. Wan et al.’s^40^ research also showed that the injectable adhesive self-healing biocompatible hydrogel has good antiadhesion performance which was attributed to its strong barrier properties (to block contact between potential inflammatory factors and the wound surface and alleviate the inflammatory response). Liang et al.^41^ developed asymmetric hydrogel (Janus hydrogel) using poly(acrylic acid), gelatin, and hyperbranched polymers modified with catechol materials. In vivo animal experiment results show that the Janus hydrogel could completely seal the surgical site and could promote the healing process of the perforation through accelerating the transition of inflammation to proliferation phase (by CD68, PCNA, and CD31 detection), with reduced risk of postoperative tissue adhesion. The above two pieces of research showed that the material regulates the inflammatory response at the surgical site to reduce the occurrence of PA.

Endogenous cytokines are stimulated and released in an auto- and *para*-crinic manner by tenocytes and fibroblasts following tendon injury or rupture. Proinflammatory cytokines (*IL-1β* and *TNF-α*) and inflammatory cytokines (*IL-6*) play important roles in tendon ECM synthesis. For example, *IL-6* knockout mice show poor mechanical properties for tendon healing, and *IL-1β* inhibits the synthesis of COL-I. Therefore, appropriate and timely levels of inflammatory cytokines are required for tendon healing and repair.^38,42,43^ The study by Yang et al.^22^ confirmed that the antibacterial hydrogel (CAOP/M/PL hydrogel which composed of l-arginine-modified CA and phenylboronic acid-modified oxidized dextran and then loaded with CuO_2_-coated MoS_2_ nanozyme and amphiphilic triblock copolymer PEG-PCL-PAE) enhanced wound healing and accelerated skin structure reconstruction through reduced infiltration of inflammatory cells caused by bacterial infection (H&E staining results to observe inflammatory cells).

*B. striata* (BS) is a traditional Chinese herbal medicine commonly used for anti-inflammation and hemostasis in wounds. Its bioactive compounds are mainly polysaccharides, also known as BSP. The pharmacological activities of BSP include hemostasis, antioxidant, anti-inflammation, promote cell proliferation, and anticancer etc. The anti-inflammatory mechanism is to regulate proinflammatory or inflammatory factors, such as IL-1β, IL-6, IL-8, IL-10, and TNF-α or to regulate the polarization of macrophages.^44^ For example, He et al.^45^ demonstrated that BSP promotes wound healing through inhibition of inflammatory cytokine (IL-1β, IL-6, and TNF-α) synthesis and release through the carrageenan-induced mouse paw edema model. Qiu et al.^46^ developed full-thickness wounds of rat back skin model and proved that the BDDE cross-linked BSP hydrogel could modulate the polarization of M1-type macrophages toward the M2-type and reduce the inflammatory response during the wound-healing phase to promote wound healing.

BSP was used in this study as the main anti-inflammatory bioactive ingredient to enhance cell proliferation and migration. The stable release of BSP in the CX/CXB membrane group modulated the inflammatory factors in the early stage and enhanced the proliferation and migration abilities of tenocytes in the second stage of cell proliferation, enabling the tendon repair process to be arranged as intended in the remodeling phase and COL-III to be maturely transformed into COL-I. However, the benefits of BSP are nonselective. The authors previously confirmed that BSP stimulates the proliferation of tenocytes and fibroblasts.^18^ Therefore, the authors developed a membrane with a bilayer structure consisting of an inner CXB layer and outer CX layer. Additionally, BDDE is metabolized in the body via hydrolysis into glycerol and succinic acid, which are further oxidized through the Krebs cycle.^47^ Therefore, the release of BSP from the CX/CXB membrane relies on an erosion-controlled system, that is, membrane degradation. Finally, the mechanical strength of the product itself is insufficient, which is a common problem of natural, biodegradable polymers such as CMC and BSP. Therefore, this study aimed to assist surgical suturing to avoid tissue adhesion caused by surgery and to promote tendon repair.

## Conclusions

CMC and BSP were successfully cross-linked in this study using BDDE to prepare a bilayer porous membrane (CX/CXB). The membrane exhibited biodegradability, a stable release of BSP, good biocompatibility, no cytotoxicity, and promoted the proliferation of tenocytes. The results of in vitro cell experiments and rabbit in vivo animal experiments showed that the membrane developed in this study could effectively reduce excessive inflammation in the tendon tissue and promote tenocyte proliferation. The proportion of postoperative peripheral tissue adhesion complications after surgery is still relatively high, especially in the abdomen, nerves, or tendons tissue. Therefore, the authors hope that the clinical application in the future will be able to wrap this CX/CXB membrane at the surgical site after the nerve or tendon of a patient is sutured. The membrane inhibits the inflammatory response and reduces the adhesion of surrounding tissues while promoting tissue proliferation by slowly degrading the membrane in the body and releasing BSP.

## Abbreviations

CX: carboxymethyl cellulose
CXB: carboxymethyl cellulose
COL-I: type-I collagen
PA: postoperative adhesion
FDA: Food and Drug Administration
CMC: carboxymethyl cellulose
BSP: *Bletilla striata*, OA, osteoarthritis
LPS: lipopolysaccharide
MW: molecular weight
GPC: gel permeation chromatography
FT-IR: Fourier transform infrared
NMR: nuclear magnetic resonance
SEM: scanning electron microscopy
SR: swelling ratio
Rt: room temperature
ELISA: enzyme-linked immunosorbent assay
DMEM: Dulbecco’s modified minimal essential medium
HDF: human dermal fibroblast
TRR: tendon rupture and repair
H&E: hematoxylin and eosin
ECM: extracellular matrix
WB: Western blotting
COL-III: type-III collagen
DCM: decorin
BGN: biglycan
TNMD: tenomodulin
TCPS: tissue culture polystyrene
tPA: tissue plasminogen
PAI-1: plasminogen activator inhibitor-1
HA: hyaluronic acid
PELA: polyethylene glycol
